# Superresolution microscopy with novel BODIPY-based fluorophores

**DOI:** 10.1371/journal.pone.0206104

**Published:** 2018-10-26

**Authors:** Amy M. Bittel, Isaac S. Saldivar, Nick J. Dolman, Xiaolin Nan, Summer L. Gibbs

**Affiliations:** 1 Biomedical Engineering Department, Oregon Health & Science University, Portland, Oregon, United States of America; 2 Thermo Fisher Scientific, Pittsburg, Pennsylvania, United States of America; 3 Knight Cancer Institute, Oregon Health & Science University, Portland, Oregon, United States of America; 4 OHSU Center for Spatial Systems Biomedicine, Oregon Health & Science University, Portland, Oregon, United States of America; University of California Berkeley, UNITED STATES

## Abstract

Multicolor single-molecule localization microscopy (SMLM) expands our understanding of subcellular details and enables the study of biomolecular interactions through precise visualization of multiple molecules in a single sample with resolution of ~10–20 nm. Probe selection is vital to multicolor SMLM, as the fluorophores must not only exhibit minimal spectral crosstalk, but also be compatible with the same photochemical conditions that promote fluorophore photoswitching. While there are numerous commercially available photoswitchable fluorophores that are optimally excited in the standard Cy3 channel, they are restricted to short Stokes shifts (<30 nm), limiting the number of colors that can be resolved in a single sample. Furthermore, while imaging buffers have been thoroughly examined for commonly used fluorophore scaffolds including cyanine, rhodamine, and oxazine, optimal conditions have not been found for the BODIPY scaffold, precluding its routine use for multicolor SMLM. Herein, we screened common imaging buffer conditions including seven redox reagents with five additives, resulting in 35 overall imaging buffer conditions to identify compatible combinations for BODIPY-based fluorophores. We then demonstrated that novel, photoswitchable BODIPY-based fluorophores with varied length Stokes shifts provide additional color options for SMLM using a combination of BODIPY-based and commercially available photoswitchable fluorophores.

## Introduction

Single-molecule localization microscopy (SMLM) is one of several superresolution microcopy (SRM) techniques that enable fluorescence imaging below the diffraction limit of light (~250 nm) [1, 2]. SMLM is able to achieve resolution on the order of ~10–20 nm through the use of photoswitchable fluorophores that stochastically switch between the fluorescent “on” state and the non-fluorescent “off” state [3]. Small molecules organic photoswitchable fluorophores are used with the SMLM technique most often termed stochastic optical reconstruction microscopy (STORM) [2, 4, 5], which has recently been expanded to multicolor techniques [6–8]. Multicolor SMLM requires careful selection of optimal probes, as fluorophores must exhibit minimal spectral crosstalk and be compatible with equivalent photochemical conditions to promote photoswitching [9, 10]. There are currently limited commercially available fluorophores with optimal spectral and photoswitching properties for multicolor SMLM imaging, as most commercially available photoswitchable fluorophores all have relatively short Stokes shifts (<30 nm), with an average of ~20 nm [11, 12]. Using fluorophores with short Stokes shifts in the conventional Cy3 and Cy5 imaging channels results in a lack of fluorophores that emit between 615–650 nm [2, 4, 10, 11, 13–17]. Filling this gap in spectral emission space with novel photoswitchable fluorophores would enhance multicolor SMLM imaging.

Small molecule photoswitching is driven by selection of appropriate imaging buffer conditions. Imaging buffer conditions have been thoroughly examined for the standard organic fluorophore scaffolds including cyanine, oxazine, and rhodamine and it has been demonstrated that different conditions are optimal for each fluorophore scaffold [5, 11, 18, 19]. For example, ascorbic acid (AA) and methyl viologen (MV) have been routinely used as reducing and oxidizing agents, respectively. Cyanine based fluorophores photoswitch best when there is more AA than MV [18], while oxazine based fluorophore photoswitching is improved when there are equal amounts of AA and MV [19]. Additionally, additives have been shown to further enhance photoswitching, such as tris(2-carboxyethyl)phosphine (TCEP) [20] and cyclooctatetraene (COT) [21] for cyanines, and sodium borohydride (NaBH_4_) for rhodamine and oxazine scaffolds [22]. NaBH_4_, unlike the TCEP and COT additives, is incubated with the dye and then washed out prior to adding the imaging buffer.

Notably, even with the many imaging buffer options that have been investigated for SMLM, little is known about the optimal conditions for the boron-dipyrromethene (BODIPY^™^) fluorophore scaffold. A few SMLM imaging studies have utilized BODIPY-based fluorophores with success [23, 24], however systematic investigation is warranted to facilitate their routine incorporation into SMLM studies. Understanding of optimal photoswitching buffers for BODIPY-based fluorophores could substantially improve the SMLM color palette as BODIPY-based fluorophores have the potential to provide a variety of short and long Stokes shift probes. Commercially available BODIPY fluorophores emit at various wavelengths throughout the visible range with short Stokes shifts [25], and recently a BODIPY-based fluorophore library was synthesized containing fluorophores with Stokes shifts of varying length that are optimally excited using a 561-nm laser [13], providing the opportunity to fill the spectral void in the currently available photoswitchable probes and expand the number of fluorophore options available for multicolor SMLM.

Herein, we evaluated common imaging buffers and additives for photoswitching, resulting in a total of 35 tested conditions to identify optimal photoswitching conditions for the BODIPY fluorophore scaffold using BODIPY FL as our model fluorophore. We also measured the cyanine based fluorophore, Alexa Fluor^™^ 647 (AF647) as our standard photoswitchable fluorophore with the varied imaging buffer conditions since it is one of the most widely used fluorophores for SMLM [4, 5, 11]. Through single molecule screening using a polyvinyl alcohol (PVA) isolation platform [10], we found multiple imaging buffer conditions that resulted in excellent photoswitching of both BODIPY FL and AF647. SMLM imaging of immunolabeled microtubules confirmed the optimal imaging buffer conditions for BODIPY FL, which also resulted in high quality SMLM immunolabeled microtubule images using AF647. Six novel BODIPY-based fluorophores with long Stokes shifts (>30 nm) and four novel BODIPY-based fluorophores with short Stokes shifts (<30 nm) [13] were selected for further evaluation of photoswitching and SMLM image quality. Photoswitching properties of our novel BODIPY fluorophores were compared to Alexa Fluor^™^ 568 (AF568), a widely used photoswitchable fluorophore imaged in the standard Cy3 channel [10, 16]. The novel BODIPY-based fluorophores with the most optimal photoswitching properties were selected for SMLM imaging of immunolabeled microtubules, where they were found to yield similar performance to AF568. These optimal imaging buffer conditions as well as use of our novel long Stokes shift BODIPY fluorophores will enable future multicolor SMLM studies.

## Results

### Photoswitching properties for the BODIPY and cyanine fluorophore scaffolds in varied imaging buffer

BODIPY FL was selected as the standard BODIPY fluorophore to determine the optimal imaging buffer conditions for photoswitching. To ensure that the selected conditions would facilitate multicolor SMLM, AF647 photoswitching was also evaluated in all imaging buffer conditions. Photoswitching properties were evaluated in 35 unique imaging buffer conditions, which consisted of seven redox conditions, each evaluated with five additives (Table 1) using a PVA single molecule isolation methodology [10]. All 35 imaging buffer conditions were evaluated in imaging buffers that had been deoxygenated using an enzymatic oxygen scavenging system known as GLOX (see Methods). Qualitative assessment of photoswitching was completed during image series collection for both BODIPY FL and AF647 where the varied imaging buffer conditions resulted in little to no photoswitching (-), some photoswitching (+), photoswitching throughout most of the image series collection (++) and bright photoswitching throughout the image series collection (+++) (Table 1). Notably, there were vast differences in intensity of the photoswitching in comparison to the background, where some buffers promoted higher photon output per photoswitching event. A subset of imaging buffer conditions was selected that promoted photoswitching ((+), (++) or (+++)) for both BODIPY FL and AF647 to facilitate multicolor SMLM using varied fluorophore scaffolds to stain a single sample.

**Table 1:** Imaging buffer conditions & observed photoswitching quality.

Imaging Buffer		Observed Photoswitching Quality	
*Reducing and/or Oxidizing Agent(s)*	*Additive*	*BODIPY FL*	*AF647*
**1) 500 μM MV, 500 μM AA**	**i)** 2 mM COT	++	++
	**ii)** 10 mM TCEP	+	-
	**iii)** 5 mM 3CP	++	++
	**iv)** NaBH4, 10mM for 5 min	++	++
	**v)** none	+	++
**2) 500 μM AA**	**i)** 2 mM COT	+	++
	**ii)** 10 mM TCEP	-	-
	**iii)** 5 mM 3CP	+	+
	**iv)** NaBH4, 10mM for 5 min	-	+
	**v)** none	-	++
**3) 500 μM MV**	**i)** 2 mM COT	++	++
	**ii)** 10 mM TCEP	-	+
	**iii)** 5 mM 3CP	++	+
	**iv)** NaBH4, 10mM for 5 min	-	-
	**v)** none	-	++
**4) 10 mM MEA**	**i)** 2 mM COT	+	++
	**ii)** 10 mM TCEP	-	-
	**iii)** 5 mM 3CP	++	+++
	**iv)** NaBH4, 10mM for 5 min	++	-
	**v)** none	+++	+++
**5) 100 mM MEA**	**i)** 2 mM COT	++	+++
	**ii)** 10 mM TCEP	-	+
	**iii)** 5 mM 3CP	++	+++
	**iv)** NaBH4, 10mM for 5 min	+	++
	**v)** none	+++	+++
**6) 143 mM βME**	**i)** 2 mM COT	++	++
	**ii)** 10 mM TCEP	-	++
	**iii)** 5 mM 3CP	-	+
	**iv)** NaBH4, 10mM for 5 min	+	++
	**v)** none	+++	++
**7) 5 mM Catechol**	**i)** 2 mM COT	+	+
	**ii)** 10 mM TCEP	-	-
	**iii)** 5 mM 3CP	+	-
	**iv)** NaBH4, 10mM for 5 min	-	+
	**v)** none	+	+

Observed Photoswitching Quality Definition: (-) = little/no photoswitching; (+) = some photoswitching; (++) = photoswitching throughout most of the imaging series; and (+++) = bright photoswitching throughout the imaging series.

**Fig 1:**
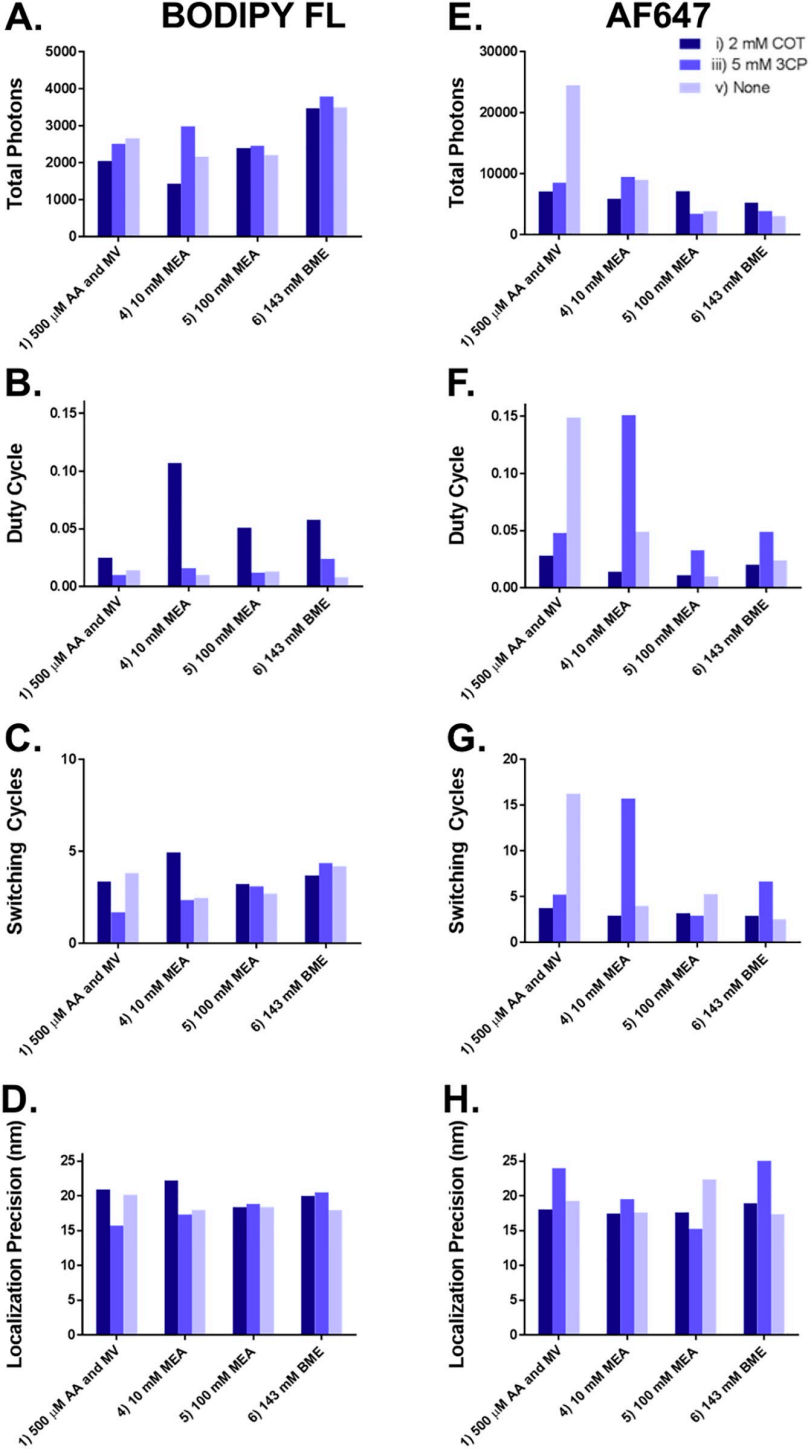
Photoswitching property quantification. The photoswitching properties of single fluorophore molecules isolated in PVA including total photon output, duty cycle, number of switching cycles and localization precision were quantified for (A)–(D) BODIPY FL and (E)–(G) AF647. The photoswitching properties were quantified in **(1)** 500 μM AA and MV, **(4)** 10 mM MEA, **(5)** 100 mM MEA and **(6)** 143 mM βME with the additives **(i)** 2 mM COT, **(iii)** 5 mM 3CP, and **(v)** no additive, results in 12 image buffer conditions tested per fluorophore.

Fluorophore photoswitching properties including total photons, duty cycle, switching cycles and localization precision were calculated for the selected image buffer conditions. Increased total photons, switching cycles and duty cycle have shown significant correlation with higher SMLM image quality, while decreased localization precision has shown significant correlation with higher SMLM image quality [10]. These four photoswitching properties were quantified for BODIPY FL and AF647 in four redox conditions including **(1)** 500 μM AA and 500 μM MV, **(4)** 10 mM 2-mercaptoethylamine HCl (MEA), **(5)** 100 mM MEA and **(6)** 143 mM 2-mercaptoethanol (βME) with three additive conditions **(i)** 2 mM COT, **(iii)** 5 mM 3-(Carboxy)-2,2,5,5-tetramethyl-1-pyrrolidinyloxy (3CP) and **(v)** no additive, resulting in 12 imaging buffer conditions (Fig 1). BODIPY FL had the highest total photon output (3,500–3,800 total photons) with redox condition **(6)** 143 mM βME using additives **(i)**, **(iii)** and **(v)** (Fig 1A). AF647 had the highest total photon output with redox conditions **(1)** 500 μm AA and 500 μM MV using additives **(i)**, **(iii)** and **(v)** (7,100–25,000 total photons) and **(4)** 10 mM MEA with the additives **(i)**, **(iii)** and **(v)** (5,900–9,600 total photons) (Fig 1E). Interestingly, no additive demonstrated a consistent influence on the total photon output for both BODIPY FL or AF647. Of note, the additive **(i)** 2 mM COT increased the duty cycle across all four redox conditions for BODIPY FL, where duty cycle was highest with imaging buffer **(4)** 10 mM MEA (Fig 1B). The additive **(iii)** 5 mM 3CP, resulted in the highest duty cycle in three out of four of the redox conditions for AF647 (Fig 1F). Likewise, BODIPY FL also had the highest number of switching cycles with the additive **(i)** 2 mM COT combined with imaging buffer **(4)** 10 mM MEA (Fig 1C), while AF647 had nearly the highest number of switching cycles with additive **(iii)** 5 mM 3CP combined with imaging buffer **(4)** 10 mM MEA (Fig 1G). Localization precision was similar across all tested imaging buffer conditions for BODIPY FL (17.9–22.2 nm) and AF647 (15.3–25.0 nm), with no consistent trends across redox reagents or additives (Fig 1D and 1H).

### Optimal imaging buffer for SMLM imaging using BODIPY and cyanine fluorophore scaffolds

**Fig 2:**
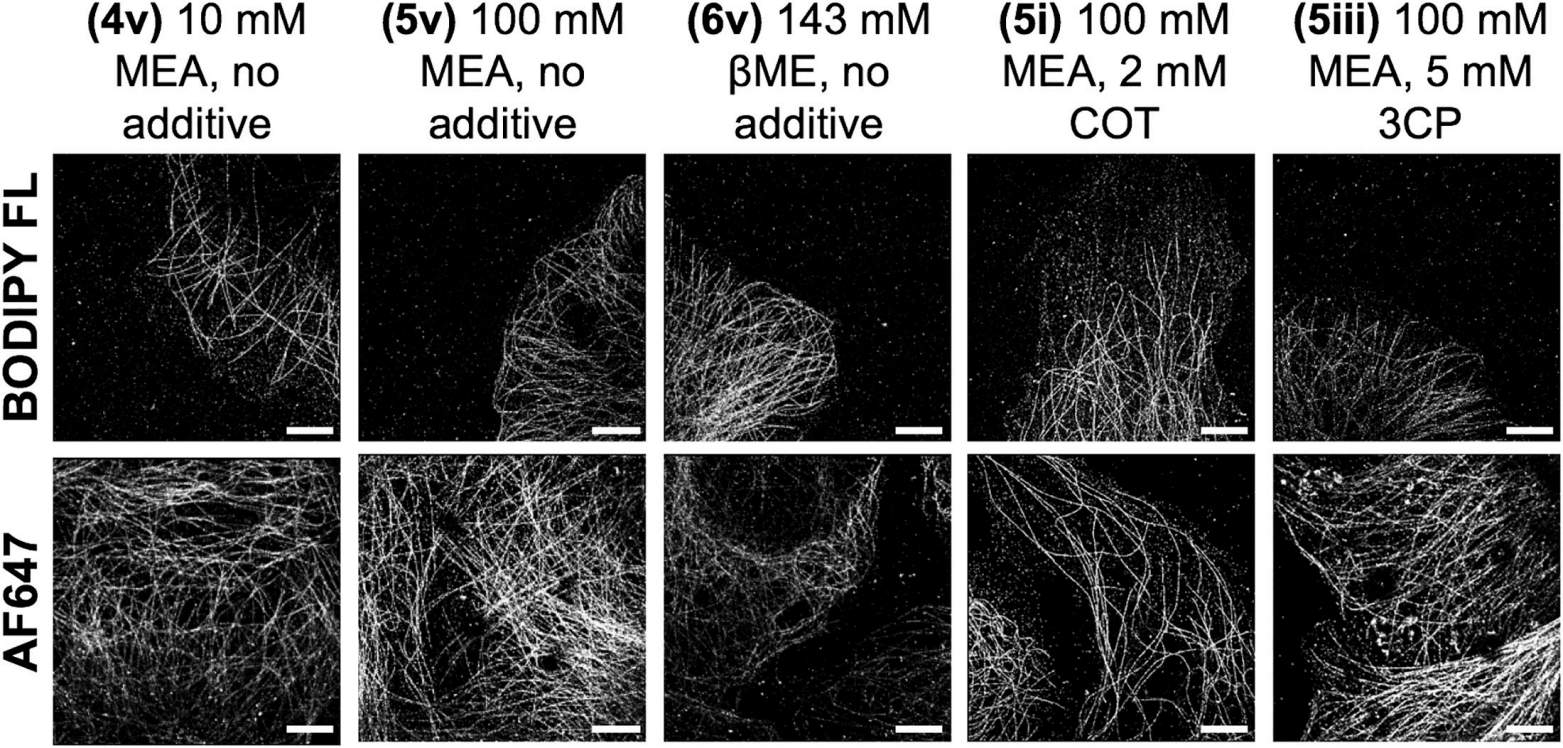
SMLM imaging of immunolabeled microtubules using a subset of imaging buffer conditions. SMLM imaging of immunolabeled microtubules *in vitro* resulted in varied image quality using BODIPY FL (top) and AF647 (bottom). Scale bar = 5 μm.

Based on the quantified photoswitching properties, a subset of five imaging buffer conditions were selected for SMLM imaging of *in vitro* immunolabeled microtubules including **(4v)** 10 mM MEA with no additive, **(5i)** 100 mM MEA with 2 mM COT, **(5iii)** 100 mM MEA with 5 mM 3CP, **(5v)** 100 mM MEA with no additive and **(6v)** 143 mM βME with no additive (Fig 2). While all imaging buffers produced images with distinct microtubule structures, there were differences in observed image quality. BODIPY FL microtubule images had the best visual contrast when using image buffer conditions **(4v)**, **(5v)** and **(6v)** and the worst contrast with image buffer conditions **(5i)** and **(5iii)**. AF647 had the highest image quality when imaged using image buffer condition **(5i)**, matching previous observations with COT [26]. However, high quality SMLM images were also rendered for AF647 using image buffer conditions **(4v)** and **(5v)** (Fig 2).

### Photoswitching properties quantification with optimal imaging buffer of novel BAA BODIPY-based fluorophores

**Fig 3:**
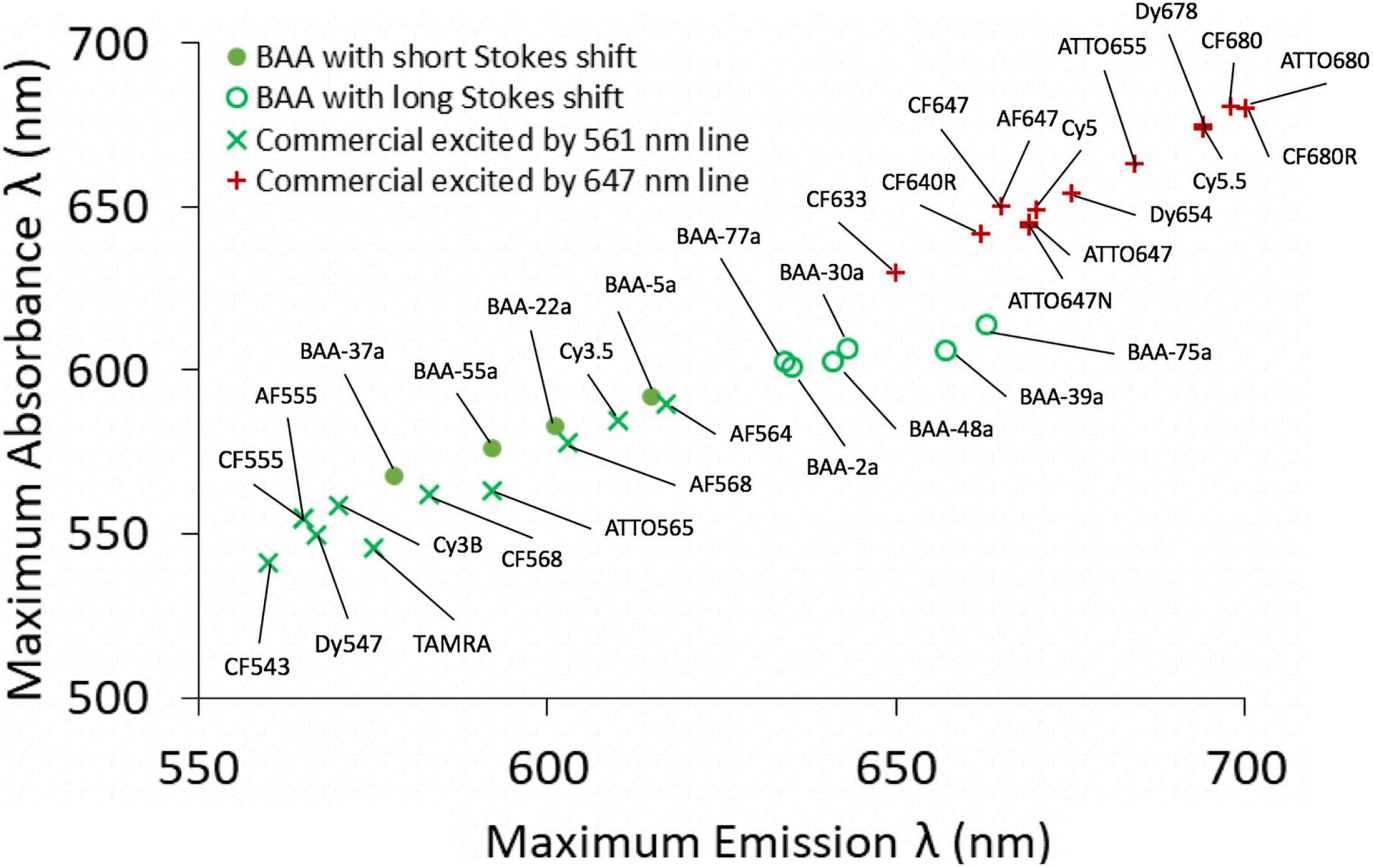
Conventional fluorophore spectral emission space gap. The maximum absorbance wavelength vs. maximum emission wavelength for conventional fluorophores excited using a standard Cy3 imaging channel/561 nm laser line (**x**) and Cy5 imaging channel/647 nm laser line (**+**) result in a gap in spectral emission space from 615–650 nm. This spectral space can be filled using novel BAA fluorophores with long Stokes shifts (○) [13]. Four additional BAA fluorophores with short Stokes shifts (●) were also evaluated for their SMLM imaging utility. Each point is labeled with the fluorophore name.

Commercially available photoswitchable fluorophores that can be imaged in the conventional Cy3 and Cy5 imaging channels all have relatively short Stokes shifts (<30 nm) with an average of ~20 nm (Table 2). This results in a lack of fluorophores that are optimally excited in the Cy3 imaging channel and emit above ~615 nm. The maximum emission of commercially available photoswitchable fluorophores that can be imaged in the conventional Cy5 imaging channel begins around 650 nm, leaving a potential 35 nm gap for addition of long Stokes shift fluorophores with optimal excitation from the Cy3 imaging channel (Fig 3 & Table 2). In an attempt to fill this spectral gap, six long Stokes shift (>30 nm) novel BODIPY-based BAA fluorophores (**BAA-39a**, **BAA-77a**, **BAA-30a**, **BAA-2a**, **BAA-48a** and **BAA-75a**) were evaluated for their utility for SMLM imaging. Additionally, four short Stokes shift (<30 nm) novel BODIPY-based BAA fluorophores (**BAA-5a**, **BAA-37a**, **BAA-22a** and **BAA-55a**) were also evaluated for their utility for SMLM imaging to compare to a commercially available photoswitchable fluorophore in the Cy3 imaging channel, AF568. Photoswitching property evaluation was performed with the optimal imaging buffer condition for the BODIPY fluorophore scaffold **(4v)** 10 mM MEA with no additive, which also enabled high quality SMLM imaging of AF647. Photoswitching properties including total photons, duty cycle, switching cycles and localization precision of single molecules isolated in PVA were quantified.

**Table 2:** Spectral properties of commercial and long Stokes shift BAA fluorophores.

Fluorophore		λ_Abs_ (nm)	λ_Em_ (nm)	Stokes shift (nm)	Laser (nm)	Reference
**Long Stokes Shift BAA FL**	BAA-77a	603	634	31	561	[23]
	BAA-2a	601	635	34	561	[23]
	BAA-48a	603	641	38	561	[23]
	BAA-30a	607	643	36	561	[23]
	BAA-39a	606	657	51	561	[23]
	BAA-75a	614	663	49	561	[23]
**Conventional Cy3 Fluorophores**	CF543	541	560	19	561	[12]
	AF555	555	565	10	561	[13]
	CF555	555	565	10	561	[12]
	Dy547	550	567	17	561	[12]
	Cy3B	559	570	11	561	[10], [11], [12]
	TAMRA	546	575	29	561	[11]
	CF568	562	583	21	561	[12]
	ATTO565	563	592	29	561	[11]
	AF568	578	603	25	561	[10], [11], [12]
	Cy3.5	585	610	25	561	[11]
	AF594	590	617	27	561	[12]
**Conventional Cy5 Fluorophores**	CF633	630	650	20	647	[12]
	CF640R	642	662	20	647	[12]
	AF647	650	665	15	647	[10], [12]
	CF647	650	665	15	647	[12]
	ATTO647	645	669	24	647	[11]
	ATTO647N	644	669	25	647	[11]
	Cy5	649	670	21	647	[10], [11]
	Dy654	654	675	21	647	[11]
	ATTO655	663	684	21	647	[11]
	Dy678	674	694	20	647	[12]
	Cy5.5	675	694	19	647	[11]
	CF680	681	698	17	647	[12]
	ATTO680	680	700	20	647	[10], [11], [12]
	CF680R	680	701	21	647	[12]
	AF680	679	702	23	647	[12]
	ATTO700	700	719	19	647	[12]
	Dy704	706	721	15	647	[12]
	AF700	702	723	21	647	[12]

**Fig 4:**
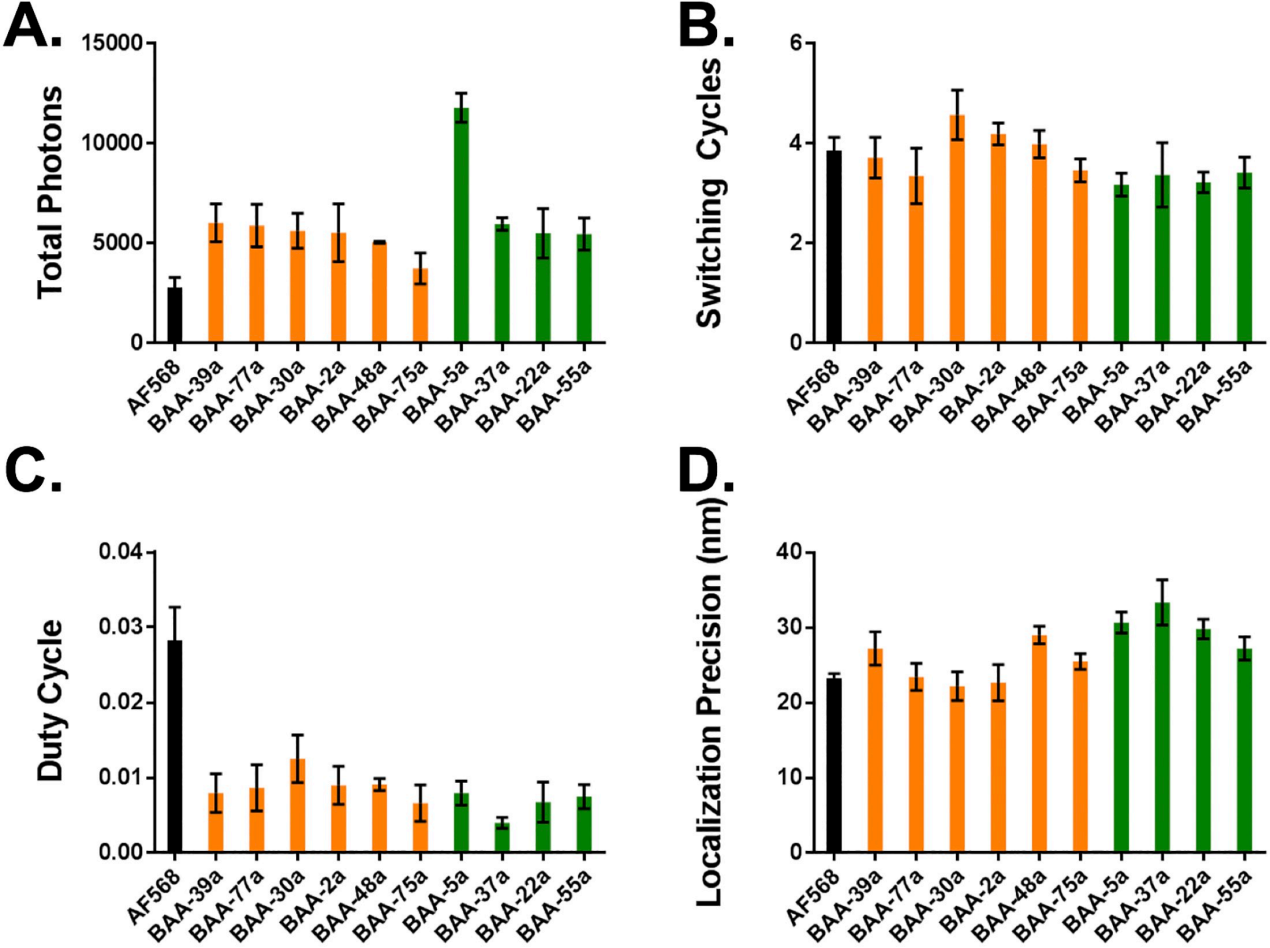
Photoswitching properties for long and short Stokes shift BAA fluorophores. Photoswitching properties including (A) total photons, (B) number of switching cycles, (C) duty cycle and (D) localization precision were quantified for AF568 (black), six BAA fluorophores with long Stokes shifts (orange), and four BAA fluorophores with short Stokes shifts (green) [13]. The average +/- standard deviation of triplicate measurements of single molecules isolated in PVA are reported.

These studies revealed that all long and short Stokes shift BAA fluorophores photoswitched and had higher total photon output than the standard Cy3 imaging channel fluorophore, AF568 (Fig 4). The long Stokes shift BAA fluorophores had lower overall photon output, (3,700–6,000 total photons) compared to the short Stokes shift BAA fluorophores (5,500–11,700 total photons). Conversely, all the BAA fluorophores had lower duty cycle than AF568. The number of switching cycles was relatively similar between the long Stokes shift BAA fluorophores (switching cycles = 3.5–4.6) and AF568 (switching cycles = 3.9), while all the short Stokes shift BAA fluorophores had fewer switching cycles (switching cycles = 3.2–3.4) than AF568. Similar localization precision was seen between AF568 (localization precision = 23.3 nm) and the long Stokes shift BAA fluorophores (localization precision = 22.2–29.0 nm), while localization precision was greater for the short Stokes shift BAA fluorophores (localization precision = 27.3–33.4 nm).

### SMLM imaging with novel BODIPY-based BAA fluorophores

A BAA fluorophore from both the long and short Stokes shift groups was selected for SMLM imaging of immunolabeled microtubules based on their quantified photoswitching properties. **BAA-30a** was selected as the long Stokes shift BAA fluorophore because of its relatively high duty cycle and number of switching cycles as well as its smaller localization precision as compared to the other long Stokes shift probes, predicting higher overall SMLM image quality. **BAA-5a** was selected as the short Stokes shift BAA fluorophore since it had the highest total photon output, which was nearly twice that of any other BAA fluorophore and a longer duty cycle than the other short Stokes shift BAA fluorophores, predicting higher SMLM image quality (Fig 4).

**Fig 5:**
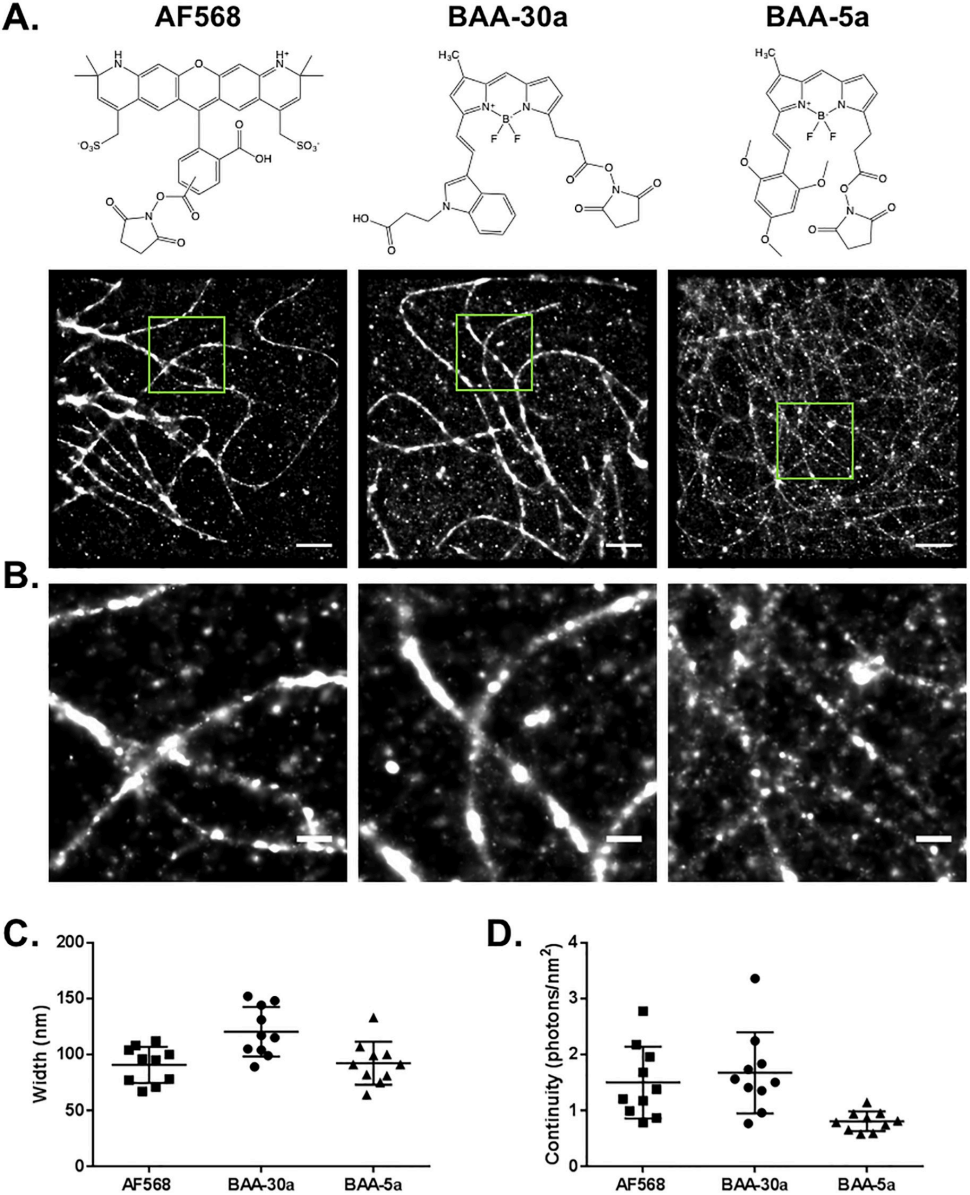
SMLM imaging with long and short Stokes shift BAA fluorophores. (A) SMLM imaging of immunolabeled microtubules *in vitro* via indirect immunofluorescence in fixed cells labeled with AF568, **BAA-30a** and **BAA-5a**, where the chemical structures of each fluorophore containing its conjugatable NHS ester group is shown. Scale bar = 2 μm. (B) Magnified images to show further detail of the boxed regions of (A) Scale bar = 0.5 μm. The average microtubule (C) width and (D) continuity for each fluorophore were calculated and are shown as the mean ± 25% of n = 10 measurements per image of the corresponding fluorophore.

As expected, both **BAA-30a** and **BAA-5a** generated SMLM images with defined microtubule structures (Fig 5A and 5B). Microtubule width and continuity were calculated for SMLM images generated using AF568, **BAA-30a**, and **BAA-5a** to quantitatively compare image quality. AF568 and **BAA-5a** had similar microtubule widths of 91 ± 16 nm and 92 ± 19 nm, respectively, and were narrower than **BAA-30a** (120 ± 22 nm). While the measured microtubule widths were wider than the expected microtubule width of 55 nm when labeled using indirect immunofluorescence [11, 26–28], they still demonstrated substantially improved resolution over conventional microscopy. Interestingly, **BAA-30a** had the highest microtubule continuity (1.67 ± 0.73 photons/nm^2^), followed by AF568 (1.50 ± 0.64 photons/nm^2^) and then **BAA-5a** (0.81 ± 0.18 photons/nm^2^). While differences were calculated in width and continuity between the fluorophores, Spearman two tail correlations revealed that these differences between AF568, **BAA-30a,** and **BAA-5a** were not significant (p>0.05), indicating that that the fluorophores performed similarly well overall for SMLM imaging (Fig 5C and 5D).

## Discussion

Multicolor SMLM is a powerful tool that will enhance our understanding of complex biological processes at the nanoscale. In order to wield the full power of this novel technology, photoswitchable fluorophores with short and long Stokes shifts that fill spectral space are required. Herein, we characterized the optimal imaging buffers for the BODIPY fluorophore scaffold to facilitate use of the long Stokes shift BODIPY-based BAA fluorophores [13] to fill known gaps in spectral space, permitting enhanced multicolor SMLM (Fig 3 and Table 2). Photoswitching properties of BODIPY FL, a well-known BODIPY based fluorophore, and AF647, the most commonly used fluorophore for SMLM were quantified in a wide range of imaging buffer conditions (Table 1). The imaging buffer that facilitated high quality SMLM images of immunolabeled microtubules using both BODIPY FL and AF647 (Fig 2) was selected for further study on the BAA long Stokes shift fluorophores to promote multicolor SMLM (Figs 4 and 5).

A total of 35 imaging buffer conditions were used to qualitatively assess BODIPY FL and AF647 photoswitching (Table 1), where a subset of 12 image buffer conditions were selected for photoswitching property quantification using a PVA single molecule isolation platform [10] (Fig 1). Higher total photon output, duty cycle and number of switching cycles has been shown to be significantly correlated with image quality, while lower localization precision has been shown to be correlated with image quality [10]. While there were a variety of imaging buffer conditions that enabled photoswitching for both BODIPY FL and AF647, there was no single imaging buffer condition and additive for either BODIPY FL or AF647 that optimized all four quantified photoswitching properties across the tested fluorophore scaffolds (Fig 1).

The five best imaging buffer conditions were selected based on quantified photoswitching properties for immunolabeled microtubule imaging using both BODIPY FL and AF647. Notably, the best SMLM images did not precisely align with the quantified photoswitching properties. For example, the imaging buffer condition **(5i)** provided the highest duty cycle and switching cycles for BODIPY FL on single molecules in PVA (Fig 1), but this did not necessarily translate to the highest SMLM image quality (Fig 2). Thus, there is a finite limitation to the single molecule PVA photoswitching screening approach, which can undoubtedly identify conditions that will produce quality images. However, the best imaging conditions cannot necessarily be identify based on photoswitching properties alone and require imaging studies for full validation. One possible explanation for this is the fact that fluorophore photoswitching is not specifically considered in the calculation of switching cycles. If photobleaching occurs during the 5,000-frame measurements the number of switching cycle is partially convolved with duty cycle. Conveniently, the best imaging buffer conditions for BODIPY FL, **(4v)** 10 mM MEA, no additive, **(5v)** 100 mM MEA, no additive and **(6v)**143 mM βME, no additive included imaging buffers commonly used with other fluorophore scaffolds such as AF647 [11, 12], making integration of BODIPY-based fluorophores into current SMLM protocols and multicolor experiments feasible (Figs 1 and 2).

Imaging buffer **(4v)** 10 mM MEA, no additive was selected for quantification of photoswitching properties of six long Stokes shift and four short Stokes shift novel BAA fluorophores [13] to evaluate their utility for SMLM. All BAA fluorophores showed higher total photon output compared to AF568, suggesting that the BAA fluorophores could provide quality SMLM images (Fig 4A). Additionally, since a greater number of switching cycles and lower localization precision are correlated with image quality [10], the relatively greater number of switching cycles and lower localization precision of the long Stokes shift BAA fluorophores could result in higher quality SMLM images than the short Stokes shift BAA fluorophores (Fig 4B and 4D). However, both the long Stokes shift and short Stokes shift BAA fluorophores had lower duty cycle than AF568 (Fig 4C), leaving it unclear after photoswitching property screening alone if the BAA fluorophores would produce improved SMLM images as compared to AF568.

**Fig 6:**
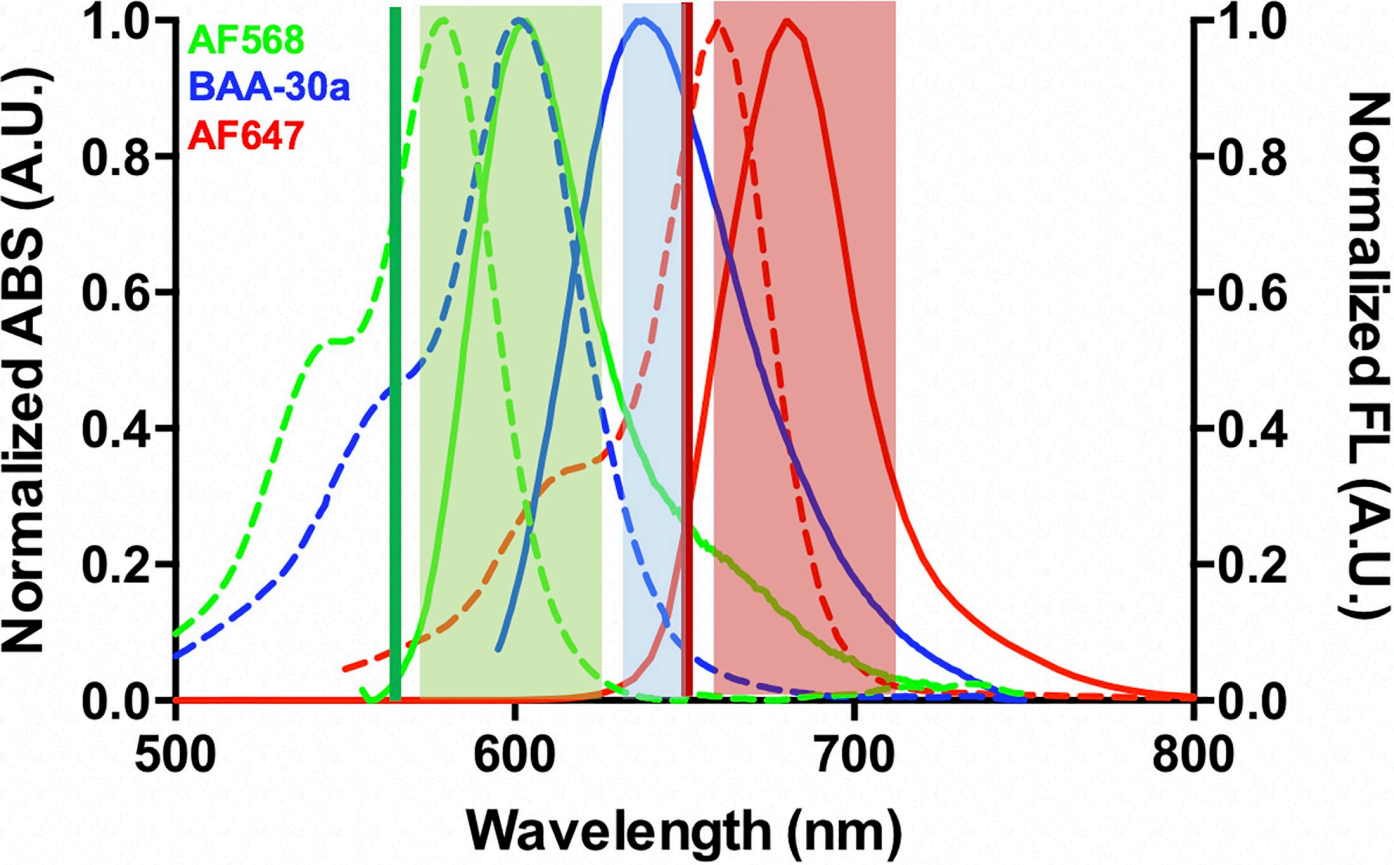
Potential optical configuration for 3-color SMLM. The normalized absorbance (dotted, right axis) and emission (solid, left axis) spectra of AF568, long Stokes shift BAA fluorophore **BAA-30a** and AF647 are shown. The 561 nm (green) and 647 nm (red) laser lines are shown with transmission of conventional Cy3 (green, 605/70 nm) and Cy5 (red, 700/75 nm) bandpass filters as well as an additional bandpass filter optimal for **BAA-30a** detection (blue, 640/20 nm) permitting detection of all three fluorophores with minimal cross talk for SMLM.

To fully assess SMLM image quality, a long Stokes shift BAA fluorophore, **BAA-30a** (emission maximum = 643 nm), and a short stokes shift BAA fluorophore, **BAA-5a** (emission maximum = 615 nm) were selected for SMLM imaging of immunolabeled microtubules *in vitro*. Both BAA fluorophores demonstrated successful immunolabeling of microtubules, confirming their utility as photoswitchable fluorophores that provide additional spectral options for multicolor SMLM (Fig 5A and 5B). While image quality varied, quantification of microtubule width and continuity demonstrated no significant difference in image quality (Fig 5C and 5D). Notably, use of **BAA-30a** provides the opportunity to create a two-color image using a single 561 nm excitation when used in combination with a standard short Stokes shift fluorophore such as AF568 or the novel **BAA-5a**. Spectral analysis of **BAA-30a** in combination with the commercially available AF568 and AF647 photoswitchable fluorophores demonstrate a potential imaging configuration for a three-color SMLM image (Fig 6). In this configuration a 561 nm laser would be used to excite both **BAA-30a** and AF568 which could be detected using optimized bandpass emission filters for two color detection. AF647 could be optimally excited using a 647 nm laser line and a conventional Cy5 bandpass filter. Little cross talk would be expected between AF647 and **BAA-30a** since minimal excitation of **BAA-30a** would be expected from the 647 nm laser and similarly, minimal excitation of AF647 would be expected from the 561 nm laser. Thus, even though there is spectral emission overlap between **BAA-30a** and AF647, sequential image acquisition should enable spectral distinction (Fig 6).

In summary, we evaluated a range of imaging buffer conditions for the BODIPY and cyanine fluorophore scaffold, where an optimal imaging buffer that facilitated SMLM imaging using both fluorophores was selected. This standard imaging buffer, **(4v)** 10 mM MEA without additive, resulted in high quality SMLM images of immunolabeled microtubules using BODIPY FL and AF647 and also facilitated high quality SMLM images using novel BODIPY-based BAA fluorophores with long and short Stokes shifts. Identification of imaging buffer conditions that result in high quality SMLM images using BODIPY-based fluorophores opens up an entire new class of fluorophores for SMLM and will permit improved multicolor SMLM imaging using long Stokes shift BODIPY-based fluorophores [13].

## Materials & methods

### Single-molecule localization microscopy (SMLM) configuration

Imaging was completed on a Nikon ECLIPSE Ti-U inverted microscope equipped with a Nikon 60x oil immersion objective (NA = 1.49) using a total internal reflection fluorescence configuration of the light path (Nikon Inc., Melville, NY). Excitation was completed using 488-nm (Coherent, Santa Clara, CA), 561-nm (Opto Engine LLC, Midvale, UT) or 647-nm lasers (Coherent), with images collected through 525/45 nm, 605/64-25 nm or 708/75 nm single-bandpass filters, (Semrock Inc., Rochester, NY), respectively [6, 10]. The 488-nm, 561-nm, and 647-nm lasers were used at illumination intensities of 0.28 kW/cm^2^, 0.49 kW/cm^2^, and 1.11 kW/cm^2^, respectively. An electron-multiplying charged-coupled device (EMCCD) camera (Andor Technology, Concord, MA) was used to record images in a 256 × 256 pixel area at 167 nm/pixel, with a 30 ms exposure time and an electron-multiplying (EM) gain setting of 300 using Micro-Manager [29, 30].

### Imaging buffers

All imaging buffers were prepared in tris-buffered saline (TN buffer, 50 mM Tris, pH 8- and 10-mM sodium chloride (NaCl)) with GLOX as the oxygen scavenger (0.5 mg/ml glucose oxidase (Sigma Aldrich, St. Louis, MO), 40 μg/ml catalase (Sigma Aldrich), and 10% w/v glucose). Seven redox conditions were tested including **(1)** 500 μM ascorbic acid (AA, Thermo Fisher Scientific, Waltham, MA) with 500 μM methyl viologen hydrate (MV, Thermo Fisher Scientific), **(2)** 500 μM AA, **(3)** 500 μM MV, **(4)** 10 mM 2-mercaptoethylamine HCl (MEA, Thermo Fisher Scientific), **(5)** 100 mM MEA, **(6)** 143 mM 2-mercaptoethanol (βME, Thermo Fisher Scientific), and **(7)** 5 mM catechol (Thermo Fisher Scientific). With each of the redox condition tested, five additives were screened including, **(i)** 2 mM cyclooctatetraene (COT, Thermo Fisher Scientific), **(ii)** 10 mM tris(2-carboxyethyl)phosphine (TCEP, Thermo Fisher Scientific), **(iii)** 5 mM 3-(Carboxy)-2,2,5,5-tetramethyl-1-pyrrolidinyloxy (3CP, Thermo Fisher Scientific), **(iv)** sodium borohydride (NaBH_4_, Sigma Aldrich), where the sample was incubated with 10 mM NaBH_4_ in Milli-Q water for 5 min, washed with TN buffer, then imaged in the remaining redox buffer, and **(v)** no additive. All imaging buffer conditions and additive combinations tested are outlined in Table 1. GLOX oxygen scavenger, redox reagent, and additives were mixed and added to the sample 10–30 min before imaging in a sealed 96 well plate with cover glass bottom (CellVis, Mountain View, CA).

### Single-molecule photoswitching analysis

BODIPY FL, Alexa Fluor 568 (AF568), and Alexa Fluor 647 (AF647) were obtained in their N-hydroxysuccinimidyl (NHS) ester form (Thermo Fisher Scientific). Novel BODIPY-based BAA fluorophores (**BAA-77a**, **BAA-2a**, **BAA-48a**, **BAA-30a**, **BAA-39a**, **BAA-37a**, **BAA-55a**, **BAA-22a**, and **BAA-5a**) were synthesized in their carboxylic acid form as described previously [13]. All NHS ester terminated fluorophores were prepared as 10 mM stock solutions in dimethyl sulfoxide (DMSO). Fluorophores were diluted to 5 nM in 1% polyvinyl alcohol (PVA, 72,000 molecular weight, MP Biomedicals, Newport Beach, CA) and 50 μl was placed per well in a 96-well glass bottom plate (Cellvis), and dried under vacuum overnight as previously described [10]. The PVA containing wells were flushed three times with 1x phosphate buffered saline (PBS) before adding imaging buffer. BODIPY FL and AF647 single-molecule photoswitching was evaluated with the 35 imaging buffer combinations. Images were collected for 5,000 frames using the 488-nm laser to optimally excite BODIPY FL and the 647-nm laser to optimally excite AF647. AF568 and the nine BAA fluorophores were only evaluated in TN buffer with GLOX and 10 mM MEA, using the 561-nm laser for optimal excitation.

Images were processed with custom written MatLab scripts (Mathworks, Natick, MA) to quantify fluorophore photoswitching properties including, total photons, duty cycle, switching cycles, and localization precision as previously described [10, 31]. Briefly, total photons represented the number of photons emitted by each molecule throughout the whole image series and was calculated as the average photons per switching cycle multiplied by the average number of switching cycles. Duty cycle represented the fraction of time each fluorophore emitted photons, with “on” frames divided by total frames. Switching cycles was the number of times a molecule changes from the “off” state to the “on” state, where it emitted photons above a set threshold. Localization precision (σ_xy_) was lateral localization precision, based on the standard deviation of the x (σ_x_) and y (σ_y_) coordinates recorded for each molecule throughout the 5,000-frame imaging video, calculated as σ_xy_ = (σ_x_^2^ + σ_y_^2^)^1/2^ [32].

### Fluorophore-labeled antibodies

The BAA fluorophores selected for SMLM imaging, **BAA-5a** and **BAA-30a**, were converted to their NHS ester functionalized form using the previously described chemistry [13]. Briefly, the carboxylic acid functionalized BAA fluorophore was diluted into dimethylformamide (DMF) and mixed with 2.2 equivalents of 1-[Bis(dimethylamino)methylene]-1H-1,2,3-triazolo[4,5-b]pyridinium 3-oxid hexafluoro-phosphate and 5.8 equivalents of diisopropylethylamine. The fluorophore mixture was activated for 30 minutes. Four equivalents of N-hydroxysulfosuccinimide was then added, followed with an additional 60 minutes of mixing. The reaction was washed with deionized water and the product was extracted with ethyl acetate before drying via lyophilization. The NHS ester functionalized versions of BODIPY FL, AF647, AF568, **BAA-5a** and **BAA-30a** were conjugated to donkey anti-rat secondary antibody (Jackson ImmunoResearch, West Grove, PA) by mixing 200 μg of antibody with the NHS ester functionalized fluorophores at a 3:1 fluorophore to antibody ratio in 1x PBS (pH 8) for 3 hours protected from light. The fluorophore antibody conjugates were purified using fast protein liquid chromatography (NGC Quest 10 Plus Chromatography System, Bio-Rad, Hercules, CA) by size exclusion (P6 gel filtration column, 40 x 12.6 mm, Bio-Rad). The fluorophore to antibody conjugation ratios were ~1–2 fluorophores per antibody, with concentrations determined using absorbance spectroscopy and fluorophore extinction coefficients (BODIPY FL = 80,000M^-1^cm^-1^, AF647 = 239,000 M^-1^cm^-1^, AF568 = 88,000 M^-1^cm^-1^, **BAA-5a** = 24,700 M^-1^cm^-1^, and **BAA-30a** = 17,600 M^-1^cm^-1^[11, 13, 25]).

### Cell culture and immunofluorescence staining

The U2OS osteosarcoma human cell line was cultured in Dulbecco’s Modified Eagle Medium without phenol red (Thermo Fisher Scientific) in 96-well glass bottom plates (Cellvis). Cells were grown to 80% confluence after which they were permeabilized and fixed with Triton-X 100 and glutaraldehyde as previously described [10]. Cells were incubated with 10 μg/ml anti-tubulin primary antibody (Jackson ImmunoResearch) for 30 minutes and washed with 1x PBS 3 times for 5 minutes per wash. The cells were then incubated with the fluorophore conjugated anti-rat secondary antibodies at 20 μg/ml protein concentration for 30 minutes protected from light. The cells were again washed 3 times for 5 minutes per wash with 1x PBS, fixed for 10 minutes with 4% paraformaldehyde, and stored in 1x PBS with 3 mM sodium azide prior to imaging.

### SMLM imaging of microtubules

Microtubules immunolabeled with BODIPY FL and AF647 were imaged for 5,000 frames at 30 Hz using five imaging buffer and additive conditions, including, **(4v)** 10 mM MEA without additive, **(5i)** 100 mM MEA with 2 mM COT, **(5iii)** 100 mM MEA with 5 mM 3CP, **(5v)** 100 mM MEA without additive and **(6v)** 143 mM βME without additive. The collected images were processed with custom written MatLab scripts [10, 31]. Constant conditions were used for processing images collected using the five selected buffering conditions. To aid in screening, single molecules were rendered at 140 nm to produce microtubule images in stark contrast to the background without optimizing conditions for particle detection. AF568, **BAA-5a** and **BAA-30a** microtubule imaging was conducted in **(4v)** 10 mM MEA without additive imaging buffer for 10,000 frames at 30 Hz. The collected images were processed with the ThunderSTORM [33] plugin for ImageJ where processing conditions were kept constant.

### Microtubule image quality quantification

The quality of the rendered SMLM images was quantified based on the previously described microtubule width and continuity [10], which were measured at 10 representative points per image where labeling was most complete and the structure was well defined. The width was calculated from line profiles summed over a 20 nm length of the microtubule structure. The continuity was calculated as the summed intensity along 0.5 μm of microtubule using a line profile 200 nm in width and was reported as photons measured per nm^2^. Spearman two-tail correlation tests were used to determine if width and continuity values were significantly different between fluorophores used for labeling, with p values less than 0.05 indicating significance.[14, 15, 17]
